# Challenges and opportunities for the next generation of computational tumor models

**DOI:** 10.1371/journal.pbio.3003269

**Published:** 2025-07-23

**Authors:** Lance L. Munn, Rakesh K. Jain

**Affiliations:** Edwin L Steele Laboratories, Department of Radiation Oncology, Massachusetts General Hospital and Harvard Medical School, Boston, Massachusetts, United States of America; Princeton University, UNITED STATES OF AMERICA

## Abstract

Mathematical models have become essential tools for exploring the complex interplay between cancer cells and their microenvironment, but require multidisciplinary expertise and abundant biological data to develop. This Perspective suggests that AI is leading the way towards the next wave of tumor models.

## Teaser

Mathematical models have become essential tools for exploring the complex interplay between cancer cells and their microenvironment, but require multidisciplinary expertise and abundant biological data to develop. AI is leading the way towards the next wave of tumor models.

**Fig 1:**
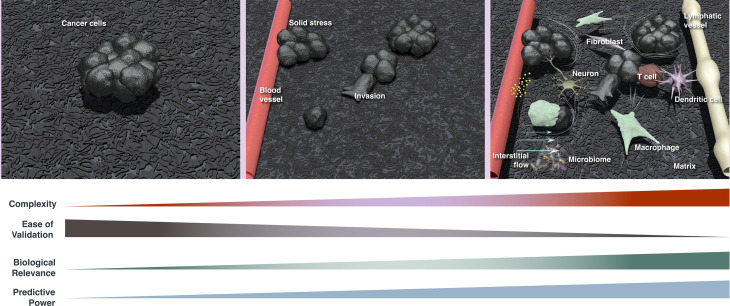
Modeling interactions and dynamics in the tumor microenvironment. Models can be focused and simple, considering only cancer cells to study, for example, cell proliferation, metabolism, mutations, etc. Adding more components and mechanisms to the model increases its complexity, but also its biological relevance and predictive power. Additional complexity requires multidisciplinary expertise, computational power and sufficient data sets to validate the model.

A solid tumor is more than a collection of cancer cells. Short-lived and rare, as well as long term, interactions between cancer cells and the tumor microenvironment (TME)—consisting of blood and lymphatic vessels, extracellular matrix, metabolites, fibroblasts, neuronal cells and immune cells (Fig 1)—govern tumor growth, progression and response to various treatments, but these events can be difficult to capture experimentally. Mathematical models have provided unprecedented insights into these interactions and have helped to improve treatments [1,2], but several barriers still remain to their widespread use.

Computational models hold great promise for hypothesis testing and virtual experimentation, as they reduce the cost, time and ethical burdens associated with in vitro, in vivo and clinical studies. By providing simulations of tumor growth, invasion and response to therapy, computational models of tumors are able to provide unique mechanistic insights into complex biological processes. Tumor models can be integrated into pharmacokinetic models to study how drugs are delivered to a tumor and how that drug affects the tumor and its environment. These hybrid quantitative systems pharmacology models can simulate diverse patient populations by varying genetic and environmental parameters, thereby facilitating virtual clinical trials and biomarker discovery [3]. Such models can predict how the spatiotemporal evolution of the TME influences therapeutic efficacy. Furthermore, computational models can be personalized using patient-specific molecular and clinical data, supporting precision oncology initiatives. By integrating multiscale data—from molecular interactions to tissue-level behaviors—they can be used to probe for interactions in the TME.

To investigate interactions within the TME, tumor cells can be modeled either as continuous populations (cell densities) or as individual agents (Fig 1). Continuous models are ideal for simulations of large cell populations, whereas individual-cell-based (agent-based) models (ABMs) allow for dynamic variation in cell phenotype, cycle, receptor levels and mutational burden, more closely mimicking biological diversity. Consequently, ABMs can capture emergent behavior (the concept that individual cells within a population, affected by local cues and cell–cell interactions, can produce unexpected, emergent multicellular phenomena) [4]. These models have the advantage of recapitulating spatial heterogeneities in the TME, in addition to capturing dynamics [5,6], and can directly incorporate simulation rules based on known mechanisms to examine synergy and emergent behavior in the contexts of metabolic competition, invasion, stem cell hierarchies and immune interactions [4].

Despite their promise, computational tumor models have not been widely adopted. Models can be tricky to validate, often owing to a scarcity of high-quality, longitudinal datasets necessary for parameter calibration and outcome benchmarking. The complexity of biologically realistic models can lead to high computational costs and scalability issues [1], yet over-simplification of models can reduce fidelity or overlook emergent behaviors. Moreover, integrating heterogeneous datasets (e.g., omics, imaging or clinical records) is technically challenging. Perhaps most importantly, constructing a biologically relevant and useful model requires knowledge of underlying biological mechanisms or rationally developed hypotheses as to how they operate. Omitting a critical mechanism can render a model non-predictive. Consequently, complex models attempting to analyze the TME generally require expertise not only from mathematicians or computer scientists, but also from oncologists, biologists, immunologists and engineers. Thus, the field of TME modeling is inherently interdisciplinary, posing practical barriers related to the establishment of collaborations for model development. Finding funding for long-term interdisciplinary modeling projects that are not immediately commercializable can also be limiting.

Advances in data science, particularly in artificial intelligence (AI) and machine learning, are opening new doors, providing more adaptability and predictive accuracy for computational tumor models [1,7,8]. Whereas mechanistic models are grounded in biological theory, AI—especially machine learning—excels at identifying patterns in high-dimensional datasets. The convergence of these paradigms has led to hybrid modeling frameworks that are more powerful and clinically applicable.

A key integration strategy involves using machine learning to complement mechanistic models by estimating unknown parameters, initializing models with multi-omics or imaging data, and reducing computational demands through surrogate modeling. For example, AI can generate efficient approximations of computationally intensive ABMs or partial differential equation models, enabling real-time predictions and rapid sensitivity analyses. Conversely, biological constraints from mechanistic models can inform AI architectures, improving model interpretability and consistency with known biology [8]. AI can also help with data assimilation and model calibration, particularly when parameters are experimentally inaccessible. Machine learning techniques can enable inference from time-series or observational data and facilitate the integration of heterogeneous datasets, including genomic, proteomic and imaging data, for robust model initialization [9–11]. Moreover, AI can contribute directly to model discovery. Symbolic regression and physics-informed neural networks can derive functional relationships and governing equations directly from data, offering new insights into tumor biology [12].

Perhaps most transformative is the use of AI-enhanced mechanistic models in clinical decision-making. The concept of patient-specific ‘digital twins’ (virtual replicas of individuals that simulate disease progression and treatment response) is becoming a reality [3]. These digital avatars integrate real-time data into mechanistic frameworks enhanced by AI, enabling personalized treatment planning, real-time monitoring and optimized therapeutic strategies tailored to individual patients. However, regulatory uncertainty regarding the acceptance and standardization of such computational modeling in clinical and pharmaceutical settings poses a significant barrier. Clinician skepticism, often fueled by concerns over model complexity, interpretability and insufficient validation, can delay integration into practice. The use of patient data also raises privacy and security concerns, particularly under stringent regulations such as GDPR and HIPAA [13]. Moreover, the rapid pace of discovery in cancer biology can render existing models obsolete, necessitating continuous updates and refinement.

Mathematical modeling can be a powerful complement to experimental methods, enabling hypothesis testing and therapy optimization in scenarios where empirical data are limited. However, increasing model complexity to better mimic biological systems introduces challenges, including the need for rigorous validation and careful management of trade-offs between realism and usability. To build robust, predictive models of the TME, interdisciplinary collaborations are essential, integrating expertise in oncology, biology, mathematics, engineering and computer science. Progress also depends on high-quality experimental and clinical data for model calibration, a need even more pressing with the growing integration of AI into oncology research. Encouragingly, the rapid expansion of multi-omics datasets and advances in AI methodologies offer promising avenues to overcome these barriers, paving the way for next-generation models that are both biologically grounded and clinically relevant.

## Abbreviations

**:**
ABMs: agent-based models
AI: artificial intelligence
TME: tumor microenvironment
